# Comparative Analysis of Osseointegration Outcomes of Titanium and Zirconia Implants: A Retrospective Observational Cohort Study

**DOI:** 10.7759/cureus.110823

**Published:** 2026-06-14

**Authors:** Tejinderjit Kaur, Sreelatha Sadasivan, Riny George, Md Amin Khan, Mani Lavanya, Amit Kumar Bisen, Prashant Pillai

**Affiliations:** 1 Department of Dentistry, Guru Gobind Singh Medical College and Hospital, Faridkot, IND; 2 Department of Oral and Maxillofacial Surgery, Sri Sai College of Dental Surgery, Vikarabad, IND; 3 Department of Oral and Maxillofacial Surgery, Al-Azhar Dental College, Thodupuzha, IND; 4 Department of Oral and Maxillofacial Surgery, RKDF Dental College and Research Centre, Bhopal, IND

**Keywords:** dental implants, marginal bone loss, osseointegration, titanium, zirconium oxide

## Abstract

Introduction: Successful implant therapy depends on achieving stable and long-lasting osseointegration in the peri-implant environment. Zirconia implants have emerged as potential alternatives to titanium implants because of their favorable esthetic properties and biocompatibility. This study aimed to compare the osseointegration outcomes of titanium and zirconia posterior single-tooth implants using clinical and radiographic parameters over a 12-month follow-up period.

Materials and methods: This retrospective observational cohort study included 50 posterior single-tooth implant sites retrieved from the institutional records between January 2018 and December 2024. Patients were allocated into the titanium implant (Group T, n = 25) and zirconia implant (Group Z, n = 25) groups. Clinical and radiographic parameters, including marginal bone loss (MBL), peri-implant probing depth, modified plaque index, bleeding on probing, bone density, and radiographic bone-to-implant contact, were assessed 12 months after loading. MBL was measured using calibrated digital radiographs analyzed with ImageJ software (National Institutes of Health, Bethesda, MD, USA). Statistical analysis was performed with significance set at p < 0.05.

Results: Baseline demographic and implant-related variables were comparable between the groups, except for the submerged healing protocol, which was significantly more common in the titanium group than in the zirconia group (P = 0.023). The median MBL was 0.58 mm in the titanium group and 0.74 mm in the zirconia group, without a statistically significant difference (p = 0.118). Bleeding on probing was observed in four (16.0%) titanium implants and seven (28.0%) zirconia implants (p = 0.308). No statistically significant differences were observed in peri-implant probing depth, plaque index, radiographic bone density, or bone-to-implant contact (all p > 0.05). Multivariable regression analysis identified the baseline peri-implant bone density as a significant predictor of MBL (p = 0.029). Inter- and intra-examiner reliabilities demonstrated excellent agreement, with intraclass correlation coefficient (ICC) values exceeding 0.90.

Conclusion: Both titanium and zirconia implants demonstrated favorable osseointegration and comparable peri-implant clinical outcomes after 12 months of functional loading. Zirconia implants may represent a clinically acceptable alternative to titanium implants in selected patients requiring metal-free rehabilitation with improved esthetic outcomes.

## Introduction

Dental implants have become an established and predictable treatment modality for the rehabilitation of partially edentulous patients, demonstrating high long-term survival rates and favorable functional outcomes [1]. Among the currently available implant materials, commercially pure titanium remains the gold standard because of its excellent biocompatibility, mechanical strength, and osseointegration capacity [2]. However, titanium implants are not devoid of their limitations. Concerns regarding metal hypersensitivity, corrosion-related ion release, soft tissue discoloration in esthetically demanding regions, and increasing patient preference for metal-free restorations have stimulated interest in alternative biomaterials [3].

Zirconia implants, particularly yttria-stabilized tetragonal zirconia polycrystals (Y-TZP), have emerged as promising alternatives owing to their favorable esthetic properties, low plaque affinity, high fracture resistance, and excellent biocompatibility [4]. Patil et al. reported that single-piece zirconia implant-supported crowns demonstrated superior masticatory efficiency and patient-reported functional outcomes compared to conventional fixed partial dentures over a 12-month follow-up period [5]. Despite encouraging experimental and short-term clinical evidence, the long-term biological behavior and peri-implant tissue response of zirconia implants remain less extensively documented than those of titanium implants.

Marginal bone loss (MBL) around implants is considered to be one of the most critical indicators of implant success and peri-implant health [6]. Additional clinical parameters such as probing depth, bleeding on probing, plaque accumulation, implant stability quotient (ISQ), and implant survival rates are equally important for evaluating osseointegration and long-term prognosis [7]. Although several studies and systematic reviews have compared titanium and zirconia implants, the available evidence remains heterogeneous because of variations in implant design, loading protocols, follow-up duration, and study methodology [8,9].

Therefore, the present retrospective observational cohort study was conducted to comparatively evaluate the osseointegration outcomes of titanium and zirconia posterior single-tooth implants using clinical and radiographic parameters over a 12-month follow-up period. The primary objective was to compare MBL between titanium and zirconia implants. Secondary objectives included comparison of peri-implant clinical parameters, radiographic bone density, ISQ, and identification of factors independently associated with MBL.

## Materials and methods

Study design and setting

The present investigation was designed as a single-center retrospective observational cohort study conducted in the Department of Oral and Maxillofacial Surgery during February 2025 to May 2025, using archived clinical and radiographic records retrieved from the institutional database of RKDF Dental College and Research Centre, Bhopal, India, between January 2018 and December 2024. This study compared the clinical and radiographic osseointegration outcomes of posterior single-tooth implant-supported restorations placed using titanium implants (Group T) and zirconia implants (Group Z). Fifty implant sites were included, comprising 25 titanium implants and 25 zirconia implants.

Group allocation was based on the implant material previously selected during routine clinical treatment, according to clinician judgment and patient preference. Since all treatments were completed before the commencement of the present study, no prospective intervention or direct patient interaction was undertaken. The study protocol followed the recommendations of the Strengthening the Reporting of Observational Studies in Epidemiology (STROBE) guidelines for observational studies [10]. Ethical clearance (RKDF/DC/2025/SS-35) was obtained from the Institutional Ethics Committee prior to data retrieval, and all patient identifiers were removed during data extraction to ensure confidentiality.

Sample size estimation

Sample size estimation was performed using G*Power software version 3.1.9 (Heinrich Heine University, Düsseldorf, Germany), with MBL at 12 months as the primary outcome variable. Based on previous literature, an intergroup difference of 0.5 mm with a standard deviation of 0.6 mm was considered clinically meaningful. At a significance level of α = 0.05 and statistical power of 80%, a minimum of 23 implant sites per group was required [11]. The final target sample size was increased to 25 implants per group to compensate for incomplete records and potential exclusions during the chart review, resulting in a total sample size of 50 implant sites.

Eligibility criteria

Eligibility assessment was performed exclusively through a retrospective chart review by a calibrated examiner prior to data extraction. Adult patients aged 25-70 years who had received a single posterior implant-supported crown in the premolar or molar region were considered eligible. Only patients with adequate preoperative alveolar bone volume confirmed by cone-beam computed tomography (CBCT), availability of baseline and follow-up radiographs, and a minimum follow-up duration of 12 months post-loading were included. Patients with uncontrolled systemic disease, heavy smoking habits, parafunctional activity, active periodontal disease, history of bone grafting at the implant site, immediate implant placement, poor-quality radiographs, and incomplete clinical documentation were excluded from the study. Implant sites with missing primary stability records or unavailable 12-month radiographic evaluations were also excluded from the study.

Implant characteristics

The control group consisted of commercially pure two-piece titanium implants with moderately rough sandblasted, large-grit, acid-etched (SLA)-equivalent surfaces, including Straumann implants (Straumann Group, Basel, Switzerland) and Nobel Biocare implants (Nobel Biocare Services AG, Zürich, Switzerland). The experimental group consisted of one-piece Y-TZP implants with moderately rough surfaces, including Straumann PURE implants (Straumann Group, Basel, Switzerland) and Z-Systems zirconia implants (Z-Systems AG, Oensingen, Switzerland). Implant lengths ranged from 10 to 12 mm, and diameters ranged from 3.8 to 4.5 mm in both groups. Posterior single-unit restorations were included to standardize the occlusal loading conditions.

Examiner calibration

Prior to formal data extraction, examiner calibration was performed to ensure the reproducibility of radiographic measurements. Two independent examiners in implant dentistry and oral radiology participated in calibration exercises. Ten radiographs that were not included in the final sample were used for the calibration of MBL measurements. Radiographs were digitally calibrated using known implant dimensions, including the implant length or thread pitch. Measurements were performed from the implant shoulder to the first visible bone-to-implant contact on both the mesial and distal surfaces using the ImageJ software version 1.54 (National Institutes of Health, Bethesda, MD, USA). Each examiner independently repeated all the measurements after a washout interval of 14 days. Inter-examiner and intra-examiner reliabilities were assessed using the intraclass correlation coefficient (ICC) with a minimum acceptable threshold of 0.90.

Data collection and extraction

Clinical and radiographic data were extracted from both paper-based and electronic dental records archived at our institution. Data were categorized into demographic variables, implant and surgical variables, radiographic measurements, and clinical follow-up parameters. The demographic variables included patient age, sex, smoking status, and systemic medical history. Implant-specific variables included implant material, dimensions, surface characteristics, healing protocol, surgical approach, insertion torque values, and stability quotient values obtained through resonance frequency analysis.

**Figure 1:**
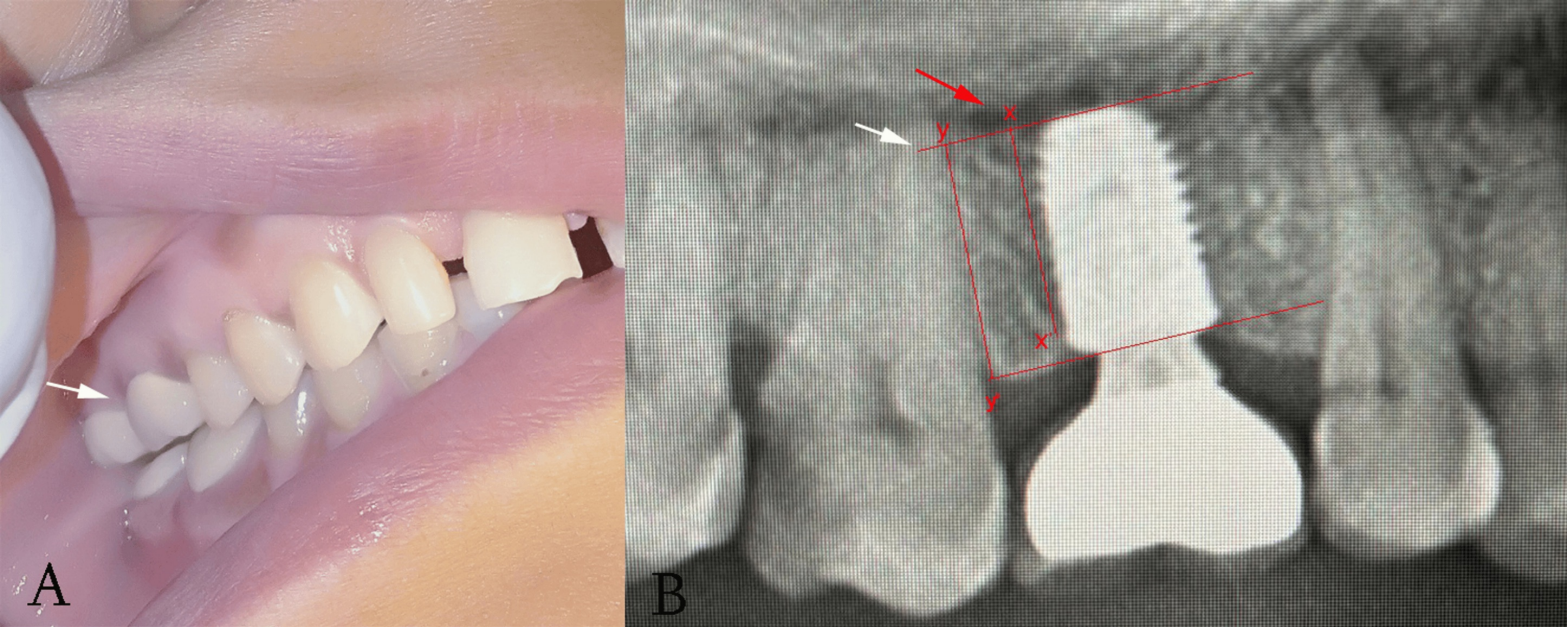
Radiographic findings. Dental implant at the maxillary right first molar showing (A) the clinical position of the gingival margin (white arrow) and (B) measurement of implant length (white arrow) and marginal bone level (red arrow). y-y' : implant length (in mm), x-x' : marginal bone height (in mm) Image analysis was performed using ImageJ software (Version 1.54, National Institutes of Health, Bethesda, MD, USA). All measurements were obtained after image calibration and recorded in the designated units of measurement.

Radiographic data were collected from standardized periapical radiographs or orthopantomograms obtained at baseline (T0) and at 12 months post-loading (T12). All radiographic analyses were performed using the ImageJ software after digital calibration. MBL was measured as the distance from the implant reference point to the first visible bone-to-implant contact on both mesial and distal aspects (Figure 1). The mean values were calculated and recorded to the nearest 0.1 mm. Where preoperative CBCT records were available, peri-implant bone density was recorded in Hounsfield units (HU). The clinical follow-up parameters recorded at 12 months included peri-implant probing depth, modified plaque index [12], bleeding on probing, implant mobility, and prosthetic complications. Implant success was assessed, while implant failure was defined as implant mobility, severe radiographic bone loss, or implant removal [13]. The follow-up record form has been attached in Appendix 1.

Outcome measures

The primary outcome measure of the study was MBL 12 months after prosthetic loading. The secondary outcome measures included peri-implant probing depth, modified plaque index, bleeding on probing, peri-implant bone density, radiographic bone-to-implant contact, and ISQ. Peri-implant bone density measurements were obtained from CBCT-derived calibrated grayscale values provided by the imaging software under standardized acquisition settings. These values were used for relative intra-study comparison between groups rather than absolute medical CT-equivalent bone density assessment. Radiographic bone-to-implant contact percentage was estimated from calibrated two-dimensional radiographic images by calculating the proportion of the implant surface appearing in direct radiographic continuity with surrounding bone. This parameter was used as a comparative radiographic indicator rather than a histological measurement of true bone-to-implant contact.

Statistical analysis

All statistical analyses were performed using the IBM SPSS Statistics (version 26.0; IBM Corp., Armonk, NY, USA) and R software version 4.3.2 (R Foundation for Statistical Computing, Vienna, Austria). Continuous variables are expressed as mean ± standard deviation for normally distributed data and median with interquartile range for non-normally distributed variables. Distribution normality was assessed using the Shapiro-Wilk test, together with visual evaluation of histograms and Q-Q plots. Intergroup comparisons for normally distributed variables were performed using the independent samples t-test, whereas non-normally distributed variables were analyzed using the Mann-Whitney U test. Categorical variables were compared using Pearson’s chi-squared test or Fisher’s exact test, as appropriate. Multivariable linear regression analysis was performed to identify the independent predictors of MBL after adjustment for clinically relevant covariates. Reliability statistics were calculated using ICCs. Statistical significance was set at p < 0.05, and the Bonferroni correction was applied for multiple secondary outcome comparisons.

## Results

Fifty posterior single-tooth implant sites were included in the study, comprising 25 (50.0%) titanium implants and 25 (50.0%) zirconia implants. The baseline demographic and clinical variables were generally comparable between the two groups (Table 1). Fourteen (56.0%) and 12 (48.0%) patients in the titanium and zirconia groups were male. Seven (28.0%) and six (24.0%) patients, respectively, had a smoking history. Controlled diabetes was documented in seven (28.0%) patients with titanium implants and eight (32.0%) patients with zirconia implants. No statistically significant intergroup differences were observed in age, smoking status, peri-implant bone density, implant dimensions, insertion torque, or ISQ values at placement (all p > 0.05). Submerged two-stage healing was significantly more frequent among titanium implants than among zirconia implants (p = 0.023).

**Table 1:** Baseline demographic, clinical, and surgical characteristics of the study groups. Data are presented as mean ± standard deviation or number. An independent samples t-test was used for normally distributed continuous variables, and a Pearson chi-square test was used for categorical variables; *p < 0.05 was considered statistically significant. SD: standard deviation, HU: Hounsfield units, ISQ: implant stability quotient, HbA1c: glycated hemoglobin

Variable		Group T - Titanium (n = 25)	Group Z - Zirconia (n = 25)	Test value	p-value
Patient characteristics	Age at placement (years), mean ± SD	42.3 ± 9.1	44.6 ± 8.7	t = -0.91	0.365
	Sex, Male n (%)	14 (56.0%)	12 (48.0%)	χ² = 0.32	0.571
	Smoking status, n (%)	7 (28.0%)	6 (24.0%)	χ² = 0.10	0.747
	Peri-implant bone density at placement (HU), mean ± SD	612.0 ± 142.0	598.0 ± 155.0	t = 0.33	0.740
	HbA1c available (controlled diabetes), n (%)	7 (28.0%)	8 (32.0%)	χ² = 0.09	0.758
Implant specifications	Implant length (mm), mean ± SD	11.2 ± 0.7	10.9 ± 0.8	t = 1.41	0.165
	Implant diameter (mm), mean ± SD	4.1 ± 0.3	4.0 ± 0.3	t = 1.17	0.244
	Insertion torque (Ncm), mean ± SD	32.4 ± 6.8	29.7 ± 7.2	t = 1.36	0.179
	ISQ at placement, mean ± SD	68.4 ± 6.2	66.1 ± 7.0	t = 1.23	0.225
Surgical protocol	Flapped approach, n (%)	21 (84.0%)	20 (80.0%)	χ² = 0.13	0.713
	Submerged (two-stage) healing, n (%)	18 (72.0%)	10 (40.0%)	χ² = 5.19	0.023*
	Posterior molar site, n (%)	16 (64.0%)	15 (60.0%)	χ² = 0.08	0.771

At the 12-month evaluation, both implant systems demonstrated favorable clinical and radiographic outcomes (Table 2). The median MBL was slightly greater in the zirconia group than in the titanium group; however, the difference was not statistically significant (p = 0.118). Similarly, the radiographic bone density and bone-to-implant contact percentages were comparable between the groups (p > 0.05). Clinical peri-implant parameters, including probing depth and modified plaque index, also showed no statistically significant intergroup differences. Bleeding on probing was observed in four (16.0%) titanium implant sites and seven (28.0%) zirconia implant sites, without a significant association (p = 0.308). Following Bonferroni correction for secondary outcomes, none of the assessed clinical or radiographic parameters showed statistically significant differences between implant materials.

**Table 2:** Comparison of changes in outcome variables between the study groups. Data are presented as mean ± standard deviation, median (interquartile range), or number. T0 indicates baseline at implant placement, and T12 indicates 12-month follow-up. An independent samples t-test was used for normally distributed continuous variables, a Mann-Whitney U test was used for non-normally distributed variables, and a Pearson chi-square test was used for categorical variables. The Bonferroni-corrected significance threshold for secondary outcomes was p < 0.008. SD: standard deviation, IQR: interquartile range, HU: Hounsfield units

Outcomes (Change from baseline T0-T12)	Measure	Group T - Titanium	Group Z - Zirconia	Test statistic	p-value
Marginal bone loss, mm	median (IQR)	0.58 (0.38-0.87)	0.74 (0.49-1.10)	U = 252.0	0.118
Bone density (HU)	mean ± SD	745 ± 85	712 ± 92	t = 1.31	0.196
Bone‑implant contact (Radiographic), %	mean ± SD	68.5 ± 12.3	63.2 ± 14.1	t = 1.42	0.162
Probing depth, mm	median (IQR)	2.8 (2.4-3.1)	3.0 (2.6-3.4)	U = 265.0	0.342
Modified plaque index (0‑3)	mean ± SD	0.62 ± 0.35	0.71 ± 0.42	t = -0.84	0.406
Bleeding on probing	n (%)	4 (16.0%)	7 (28.0%)	χ² = 1.04	0.308

Multivariable linear regression analysis identified peri-implant bone density at placement as a significant independent predictor of MBL at 12 months (p = 0.029) (Table 3). Increased baseline bone density was associated with reduced MBL. The implant material showed a non-significant trend toward higher bone loss in the zirconia group (p = 0.067). Other covariates, including patient age, implant diameter, and healing protocol, were not significantly associated with MBL. The regression model explained 31.2% of the total variance in MBL measurements.

**Table 3:** Multivariable linear regression analysis for predictors of marginal bone loss at 12 months. Dependent variable: marginal bone loss at 12 months (mm). Multivariable linear regression analysis was performed to identify independent predictors; p < 0.05 was considered statistically significant. *Indicates statistically significant predictor. SE: standard error, CI: confidence interval, HU: Hounsfield units, VIF: variance inflation factor

Predictor variable	β (Unstandardized)	SE	β (Standardized)	95% CI	p-value
Implant material: Zirconia vs. Titanium (reference)	0.183	0.097	0.271	-0.014 to 0.380	0.067
Patient age (per year)	0.008	0.005	0.228	-0.002 to 0.018	0.121
Bone density at placement (per 100 HU)	-0.041	0.018	-0.312	-0.078 to -0.004	0.029*
Implant diameter (per mm)	-0.124	0.093	-0.189	-0.314 to 0.066	0.189
Healing protocol (submerged/non-submerged)	-0.142	0.081	-0.214	-0.306 to 0.022	0.089
Model intercept	1.042	0.384	-	0.263 to 1.821	0.010*
Model fit: R² = 0.312; Adjusted R² = 0.231; F(5,44) = 3.99; p = 0.005					

Reliability analysis demonstrated excellent reproducibility of the radiographic measurements (Table 4). Inter-examiner agreement yielded an ICC of 0.94, while intra-examiner ICC values were 0.96 and 0.95 for Examiner A and Examiner B, respectively, indicating excellent consistency and measurement reliability throughout the study period.

**Table 4:** Examiner reliability statistics using intraclass correlation coefficients. Intraclass correlation coefficient was estimated using a two-way mixed-effects model with absolute agreement and single-measures configuration; ICC ≥ 0.90 was considered acceptable reliability. CI: confidence interval, ICC: intraclass correlation coefficient

Intraclass correlation coefficient (ICC)	ICC	95% CI	Interpretation
Inter-examiner reliability (Examiner A vs. Examiner B)	0.94	0.88 - 0.97	Excellent (≥0.90 threshold met)
Intra-examiner reliability - Examiner A	0.96	0.91 - 0.98	Excellent (≥0.90 threshold met)
Intra-examiner reliability - Examiner B	0.95	0.90 - 0.97	Excellent (≥0.90 threshold met)

## Discussion

This retrospective observational cohort study compared the osseointegration outcomes of titanium and zirconia posterior single-tooth implants over a 12-month follow-up period using clinical and radiographic parameters. The findings demonstrated that both implant systems exhibited favorable peri-implant tissue health and comparable osseointegration characteristics, with no statistically significant differences in MBL, peri-implant probing depth, plaque accumulation, bleeding on probing, or radiographic bone-to-implant contact. These findings indicate that contemporary zirconia implants may achieve a biological performance similar to that of titanium implants when placed under appropriate clinical conditions.

MBL is considered one of the most important indicators of implant success because it reflects the stability of the peri-implant hard tissue environment following functional loading [6]. In the present study, zirconia implants demonstrated a slightly greater median MBL than titanium implants, although the difference was not statistically significant. One possible explanation for this finding could be related to differences in implant design and healing protocols. Most zirconia implants included in the present study were one-piece systems restored using a non-submerged healing approach, whereas titanium implants more frequently follow a two-stage submerged protocol. Submerged healing may reduce early occlusal micromovement and bacterial contamination during osseointegration, thereby contributing to slightly lower crestal bone remodeling around titanium implants. Nevertheless, the observed MBL values in both groups remained within the acceptable physiological range proposed by Albrektsson et al., indicating successful peri-implant adaptation during the first year of loading [13].

The absence of a statistically significant difference in MBL between the groups is consistent with previous investigations comparing titanium and zirconia implants. In a recent systematic review and meta-analysis, Peña-Cardelles et al. reported similar survival rates and peri-implant bone loss values between titanium and titanium-zirconia implants [11]. Similarly, Roehling et al. observed that zirconia implants demonstrated osseointegration patterns and bone healing responses comparable to those of titanium implants in both experimental and clinical settings [14]. The comparable bone response observed in the present study may be attributed to improvements in zirconia surface engineering, including sandblasted and acid-etched moderately rough surfaces that enhance osteoblast attachment and bone maturation.

The peri-implant soft tissue findings of the present study also demonstrated no significant differences between the groups regarding probing depth, plaque index, and bleeding on probing. These observations may be explained by the favorable soft tissue compatibility and low bacterial adhesion properties of zirconia surfaces. Previous in vitro and clinical studies have demonstrated reduced plaque accumulation on zirconia compared to titanium because zirconia possesses lower surface free energy and smoother surface characteristics, thereby limiting bacterial colonization [14,15]. Osman and Swain reported that zirconia surfaces may favor improved peri-implant mucosal health owing to their low affinity for oral biofilm formation [15]. In the present study, although bleeding on probing was numerically higher in zirconia implants, the difference was not statistically significant, possibly because all the included patients maintained acceptable oral hygiene and regular follow-up care.

Radiographic bone density and bone-to-implant contact percentages were also comparable between the implant systems. This finding suggests that zirconia implants can achieve adequate biomechanical integration and functional stability, similar to that of titanium implants. Modern zirconia materials, particularly Y-TZP, possess high flexural strength and excellent biocompatibility, which contribute to favorable osseointegration [16]. Andreiotelli et al. suggested that zirconia implants may represent a clinically acceptable alternative to titanium implants, especially in patients requiring improved aesthetics or metal-free rehabilitation [17].

An important finding of the present study was the significant association between the baseline peri-implant bone density and MBL at 12 months. Higher bone density at implant placement is associated with reduced peri-implant bone loss, indicating that host bone quality may exert a greater influence on peri-implant remodeling than the implant material itself. Dense cortical bone generally provides superior primary stability and a more favorable load distribution during healing and functional loading, thereby reducing crestal stress concentration and bone resorption [18]. In contrast, the implant material did not remain a statistically significant independent predictor after multivariable adjustment, further supporting the concept that both titanium and zirconia implants possess comparable biological integration potential under favorable bone conditions.

The excellent intra- and inter-examiner reliability demonstrated in the present study further strengthens the validity of the findings. ICC values exceeding 0.90 indicated high reproducibility of radiographic measurements and minimized the likelihood of observer-related bias. The use of calibrated digital radiographic assessment using ImageJ software also improved measurement precision and standardization.

From a clinical perspective, the findings of the present study suggest that zirconia implants may serve as predictable alternatives to titanium implants for posterior single-tooth rehabilitation. Their favorable aesthetic properties, absence of metallic color transmission, and potential for reduced plaque accumulation may provide additional advantages for patients with thin gingival biotypes, metal sensitivity concerns, or a preference for metal-free restorations. However, clinicians should be cautious regarding the mechanical limitations of one-piece zirconia implant systems, including reduced prosthetic flexibility and increased technical sensitivity during placement and restoration.

Certain limitations of the present study should be acknowledged. The retrospective cohort design may have introduced selection bias and limited control over potential confounding variables. The relatively small sample size and single-center setting may reduce the external generalizability of the findings. Additionally, the 12-month follow-up duration may not adequately reflect long-term peri-implant tissue stability or late biological and prosthetic complications. Although the CBCT system used in the study provided calibrated density values expressed in HU units, CBCT-derived density measurements may vary depending on scanner characteristics, acquisition parameters, and reconstruction algorithms; therefore, these values should primarily be interpreted as relative intra-study radiographic density estimates rather than absolute medical CT-equivalent HU. Furthermore, the inclusion of different implant systems, implant designs, prosthetic protocols, surface characteristics, implant-abutment connection configurations, and healing approaches within the titanium and zirconia groups may have introduced methodological heterogeneity that could have influenced peri-implant tissue responses and osseointegration outcomes. Because implant-associated variables were assessed retrospectively from archived clinical records, complete standardization of surgical techniques, occlusal loading conditions, prosthetic maintenance protocols, and follow-up documentation could not be ensured. In addition, radiographic bone-to-implant contact assessment was based on two-dimensional radiographic interpretation rather than histological evaluation, which may limit precise quantification of true osseointegration. Moreover, because of the retrospective nature of the study, clinical and radiographic parameters were dependent on the completeness and standardization of archived institutional records, which may have introduced variability in documentation and follow-up assessment. Therefore, further multicenter prospective randomized controlled trials with larger sample sizes, standardized implant systems, and extended follow-up durations are recommended to establish stronger evidence regarding the long-term comparative performance of titanium and zirconia implants.

## Conclusions

Within the limitations of this retrospective observational cohort study, both titanium and zirconia implants demonstrated favorable osseointegration outcomes and satisfactory peri-implant tissue health over a 12-month follow-up period. No statistically significant differences were observed between the two implant systems in terms of MBL, peri-implant clinical parameters, or radiographic bone integration. Baseline peri-implant bone density has emerged as a significant factor influencing marginal bone remodeling, irrespective of the implant material. These findings suggest that zirconia implants may represent a clinically acceptable alternative to conventional titanium implants, particularly in patients seeking metal-free and aesthetically favorable restorative options. However, long-term prospective studies are required to validate these outcomes further.

## Figures and Tables

**Figure 2:**
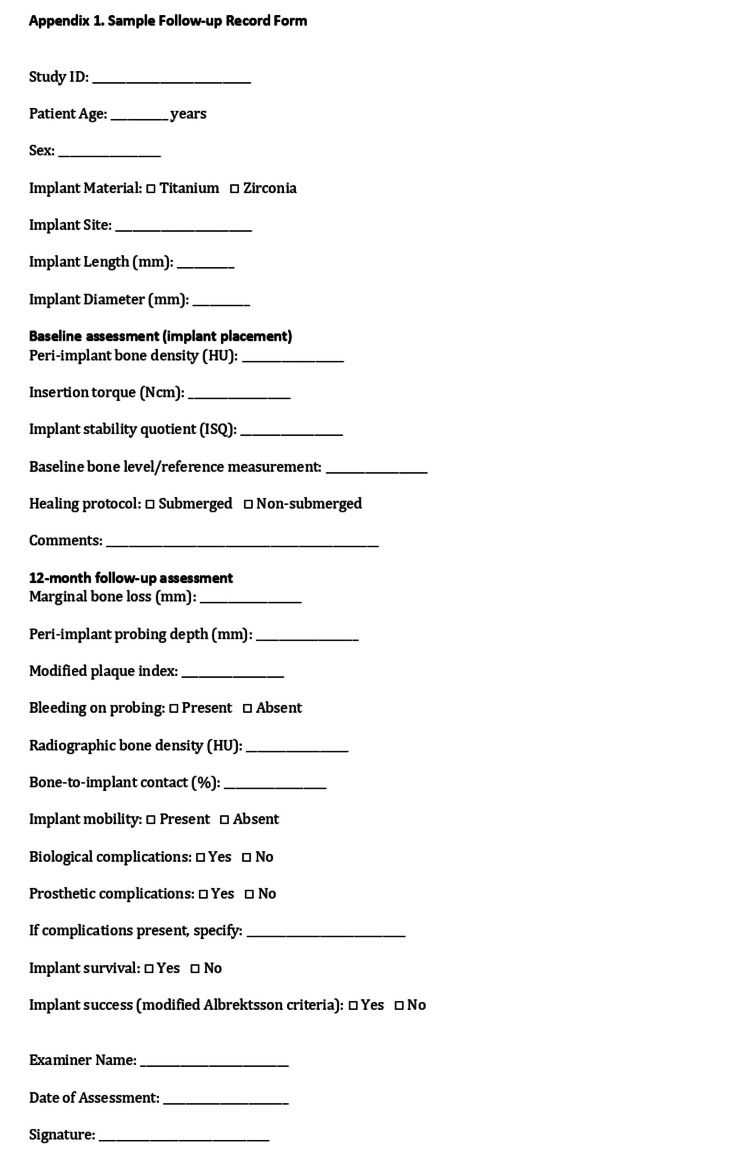
Follow-up record form used in the study.

## Appendices

Appendix 1 
